# Tumor-associated antigen prediction using a single-sample gene expression state inference algorithm

**DOI:** 10.1016/j.crmeth.2024.100906

**Published:** 2024-11-18

**Authors:** Xinpei Yi, Hongwei Zhao, Shunjie Hu, Liangqing Dong, Yongchao Dou, Jing Li, Qiang Gao, Bing Zhang

**Affiliations:** 1 Department of Bioinformatics and Biostatistics, School of Life Sciences and Biotechnology, Shanghai Jiao Tong University, Shanghai 200240, China; 2 Lester and Sue Smith Breast Center, Baylor College of Medicine, Houston, TX 77030, USA; 3 Department of Molecular and Human Genetics, Baylor College of Medicine, Houston, TX 77030, USA; 4 Department of Liver Surgery and Transplantation, Liver Cancer Institute, Zhongshan Hospital and Key Laboratory of Carcinogenesis and Cancer Invasion of the Ministry of China, Fudan University, 180 Fenglin Road, Shanghai 200032, China

**Keywords:** tumor-associated antigen, Bayesian algorithm, gene expression state inference, liver cancer, immunotherapy, therapeutic target, pan-cancer, CAR-T, TCGA, CPTAC

## Abstract

We developed a Bayesian-based algorithm to infer gene expression states in individual samples and incorporated it into a workflow to identify tumor-associated antigens (TAAs) across 33 cancer types using RNA sequencing (RNA-seq) data from the Genotype-Tissue Expression (GTEx) and The Cancer Genome Atlas (TCGA). Our analysis identified 212 candidate TAAs, with 78 validated in independent RNA-seq datasets spanning seven cancer types. Eighteen of these TAAs were further corroborated by proteomics data, including 10 linked to liver cancer. We predicted that 38 peptides derived from these 10 TAAs would bind strongly to HLA-A02, the most common HLA allele. Experimental validation confirmed significant binding affinity and immunogenicity for 21 of these peptides. Notably, approximately 64% of liver tumors expressed one or more TAAs associated with these 21 peptides, positioning them as promising candidates for liver cancer therapies, such as peptide vaccines or T cell receptor (TCR)-T cell treatments. This study highlights the power of integrating computational and experimental approaches to discover TAAs for immunotherapy.

## Highlights

• Bayesian algorithm infers gene expression states in individual samples
• 212 candidate TAAs found across 33 cancer types using GTEx and TCGA data
• 78 TAAs validated in independent RNA-seq datasets spanning seven cancer types
• Experimental validation confirms 21 immunogenic peptides from 10 liver cancer TAAs

## Motivation

Tumor-associated antigens (TAAs), which are scarcely present in normal tissues but notably elevated in tumors, hold significant promise for immunotherapy. Gene expression databases such as Genotype-Tissue Expression (GTEx) for normal tissues and The Cancer Genome Atlas (TCGA) for tumor tissues enable the systematic evaluation of gene expression in both normal and cancerous conditions, facilitating the identification of TAAs. Some statistical methods focus on tissue-level gene expression state inference, limiting their utility in identifying specific tumors for personalized treatments. Conversely, single-sample analysis relying on a fixed threshold method struggles to effectively handle measurement noise.

## Graphical abstract

**Figure:**
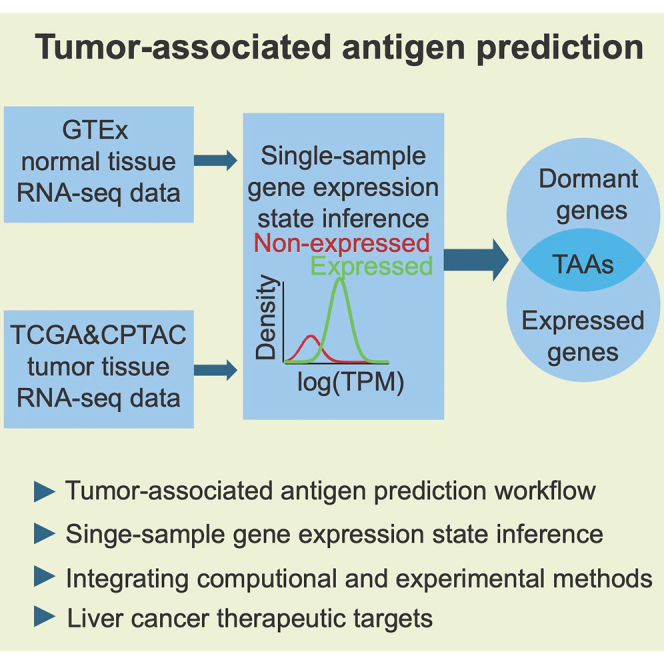

## Teaser

Yi et al. present a Bayesian algorithm to infer gene expression states in individual samples, identifying 212 tumor-associated antigens (TAAs) across 33 cancer types. Experiments confirmed 21 immunogenic peptides from 10 liver cancer TAAs. Notably, 64% of liver tumors express these TAAs, highlighting their potential as candidates for immunotherapy development.

## Introduction

Tumor immunotherapy has emerged as an indispensable component of modern cancer treatment.^1^ The success of such therapies largely hinges on the ability of T cells to detect and respond to tumor antigens.^2^ These antigens are presented on the surface of tumor cells by the major histocompatibility complex (MHC) proteins, also known as human leukocyte antigens (HLAs) in humans. Such presentation enables the interaction between the immunogenic tumor antigens and the T cell receptor (TCR), triggering an immune response aimed at tumor eradication. Therefore, the identification of tumor antigens plays a pivotal role in developing cancer immunotherapy approaches.

Tumor antigens are categorized into two main types: tumor-specific antigens (TSAs) and tumor-associated antigens (TAAs).^3^^,^^4^^,^^5^ TSAs, such as neoantigens originating from somatic mutations, are unique to cancer cells. However, neoantigens are generally unique to each patient, which limits their widespread application.^6^^,^^7^^,^^8^ In contrast, TAAs that arise from dysregulated gene expression are more likely to be shared across patients. Some TAAs, like HER2 in breast cancer, are overexpressed in cancer cells but also present in normal tissues at lower levels. Targeting these TAAs may lead to on-target/off-tumor toxicity. Another subset of TAAs, namely the cancer/testis antigens (CTAs), are typically restricted in expression to testicular (and occasionally ovarian) germ cells in normal conditions but aberrantly expressed in various cancers. CTAs are recognized for their high specificity to cancer and the potential for broader applicability across different cancer types, making them attractive targets for immunotherapy.^9^^,^^10^ The CTA database (http://www.cta.lncc.br/) offers a manually curated collection of 276 potential CTA genes.^11^ However, it has been noted that some antigens within this database also appear in non-cancerous tissues,^12^ underscoring the urgent need to validate these candidate CTAs and uncover additional ones that may have been overlooked.

Publicly available gene expression databases such as the Genotype-Tissue Expression (GTEx)^13^ project and The Cancer Genome Atlas (TCGA)^14^^,^^15^ provide invaluable resources to tackle this challenge. GTEx provides extensive gene expression data across 54 normal tissues, whereas TCGA includes gene expression data for 33 cancer types. Collectively, these resources offer a unique opportunity to uncover genes that are either not expressed in any normal tissues or are exclusively expressed in the testis yet show aberrant expression in tumors. This enhances our ability to identify cancer-specific TAAs.

Predicting cancer-specific TAAs using gene expression data requires determining gene expression states in both tumor and normal samples. A straightforward method involves setting a fixed expression threshold to classify genes as either expressed or non-expressed. However, this method struggles to accommodate the measurement noise prevalent at lower expression levels.^16^ zFPKM^17^ is an RNA sequencing (RNA-seq) expression normalization method that identifies a threshold to distinguish between active and background gene expression. It assumes that a human cell’s transcriptome can be divided into active genes, which carry out the work of the cell, and other genes that are likely by-products of biological or experimental noise. The threshold of zFPKM = −3 was determined based on the point where the ratio of active to repressed promoters drops below 1, indicating a reasonable cutoff for distinguishing between expressed and non-expressed genes. The EnB model^18^ is another method for inferring gene expression states using bulk RNA-seq data. It estimates the frequency distribution of gene expression levels, employing an exponential distribution for transcripts from inactive genes and a negative binomial distribution for active genes for transcripts per million (TPM) RNA-seq expression. The threshold is determined at a given probability (e.g., 1%) to distinguish between non-expressed and expressed genes. Hebenstreit et al.^19^^,^^20^ have proposed that gene expression, as measured by RNA-seq, follows a bimodal distribution. This allows genes to be divided into the lowly expressed and highly expressed categories, corresponding to inactive and active states, respectively. ZigZag,^21^ a hierarchical mixture model, has been developed to capture both the bimodal distribution of gene expression levels and technical variables, allowing for accurate inference of gene expression states for individual tissue types or species. However, this method is designed for cohort-based analysis and does not extend to evaluating gene expression states in individual samples. There remains a critical need for single-sample gene expression state inference in personalized medicine contexts.

In this study, we developed a Bayesian single-sample gene expression state inference algorithm, which demonstrated greater stability and balanced performance compared to existing approaches in a benchmarking study. We incorporated this algorithm into a computational workflow to identify cancer-specific TAAs and associated tumor samples. By utilizing RNA-seq data from GTEx normal tissues and TCGA tumor samples, our workflow identified 212 candidate TAAs across 33 TCGA cancer types. Validation across seven independent cancer types confirmed 78 out of the 212 predicted TAAs, with 18 further corroborated through proteomics. Peptides derived from 10 proteomics validated that TAAs in liver cancer underwent computational analysis to evaluate their binding affinity to HLA-A02, the most prevalent HLA allele in humans. Those predicted to bind strongly to HLA-A02 were chosen for experimental validation to confirm their binding affinity and their ability to trigger an immune response.

## Results

### Computational workflow for predicting candidate TAAs

**Figure 1:**
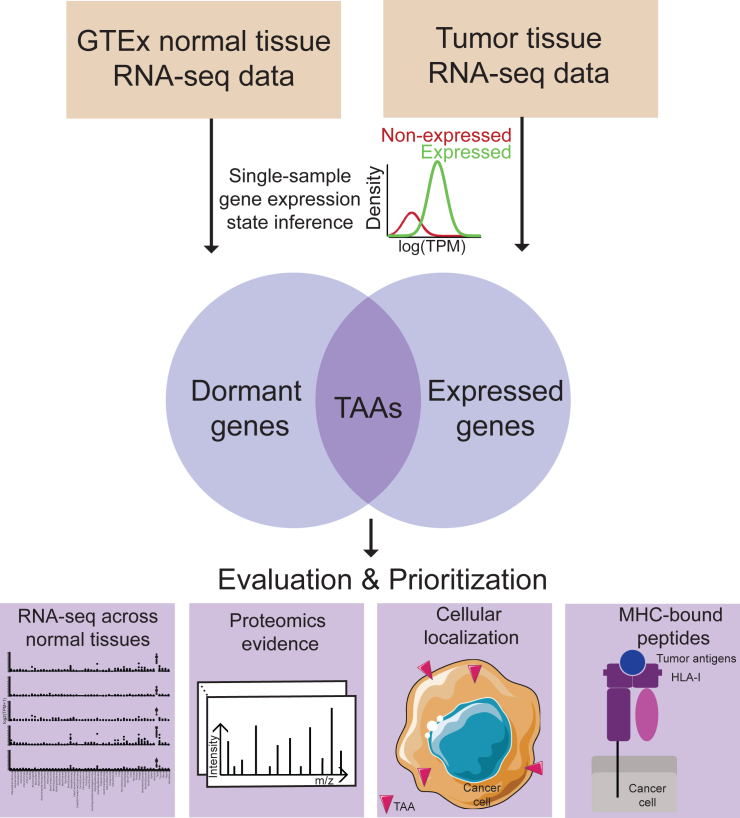
Computational workflow for predicting and prioritizing TAAs The integrated workflow comprises four steps. (1) Dormant gene prediction: single-sample expression state inference for each normal sample using all GTEx RNA-seq data, classifying genes into expressed and non-expressed states. Genes were defined as dormant genes if they showed a non-expressed ratio of at least 90% in each type of normal tissue individually, with the exception of testis. (2) Expressed genes of tumor tissue prediction: single-sample expression state inference for each tumor sample from tumor tissue RNA-seq data, classifying genes into expressed and non-expressed states. Genes with at least a 5% expressed ratio across one certain tumor tissue were defined as expressed genes. (3) TAA prediction: the overlap between dormant genes and expressed genes in tumor tissue were designated as candidate TAAs. (4) Evaluation and prioritization: visualizing RNA abundance across different normal tissues, verifying proteomics evidence, determining cellular localization, and predicting MHC-bound peptides.

To identify TAAs, we developed a computational workflow (Figure 1). This workflow utilized a Bayesian single-sample gene expression state inference algorithm, which we introduced in this study (STAR Methods). The first step in the workflow was to identify “dormant genes” in normal tissues. We started by compiling RNA-seq gene expression data from GTEx for 54 normal tissues, followed by applying our single-sample gene expression state inference algorithm to each sample individually. For each sample, our algorithm categorized all genes into expressed or non-expressed states (STAR Methods). We then aggregated the inferred gene states for each gene across different tissue types, calculating the proportion of samples in each tissue type where a gene was classified as non-expressed (STAR Methods). Genes were defined as dormant genes if they showed a non-expressed ratio of at least 90% in each type of normal tissue individually, with the exception of testis. Next, for a given RNA-seq gene expression dataset of human tumors, we applied the same gene expression state inference and classified genes as either expressed or non-expressed in individual samples (STAR Methods). Dormant genes with an expressed state in a minimum of 5% tumor samples were identified as candidate TAAs. To evaluate and prioritize these candidate TAAs, we conducted several additional analyses. These included visualizing RNA abundance across different normal tissues, verifying proteomic evidence when proteomic data were available, determining cellular localization, and predicting their potential for generating MHC-bound peptides.

### Performance evaluation of the single-sample gene expression state inference algorithms

To benchmark the performance of our single-sample gene expression state inference algorithm, we utilized chromatin epigenetic marks from the Roadmap Epigenomics Project^22^ to classify gene states in four normal tissues, liver, lung, ovary, and pancreas, as outlined in a prior study.^21^ This approach identified 9,764 activated and 5,564 inactivated genes in the liver, 9,610 activated and 5,440 inactivated genes in the lung, 10,187 activated and 5,166 inactivated genes in the ovary, and 10,207 activated and 4,177 inactivated genes in the pancreas (Table S1). By treating activated genes as expressed and inactivated genes as non-expressed, we used the epigenomics-based classifications as a reference standard to evaluate different methods for determining gene expression states in individual samples based on GTEx liver, lung, ovary, and pancreas RNA-seq gene expression data, encompassing 226, 578, 180, and 330 samples, respectively (STAR Methods).

Table 1 presents a comparison among our single-sample gene expression state inference algorithm (variable), zFPKM, EnB, and the traditional fixed threshold filtering method (TPM < 1, TPM < 2). The performance of the fixed threshold filtering method exhibited a high level of variability across different tissues and thresholds, underscoring the challenge of arbitrary threshold selection. Notably, in pancreatic tissue, both thresholds resulted in markedly low precision for detecting non-expressed genes, increasing the risk of false positives and potentially leading to on-target-off-tumor toxicity. The zFPKM and EnB methods achieved the highest recalls (0.96) in identifying expressed genes. However, EnB showed the worst overall performance among all methods in other comparisons. While zFPKM further achieved the highest precision in identifying non-expressed genes (0.98–0.99), it also demonstrated low recall for non-expressed genes (0.45–0.55) and low precision for expressed genes (0.78–0.85). Our method achieved mean precision scores ranging from 0.94 to 0.98 and mean recall scores from 0.63 to 0.71 in identifying non-expressed genes across the four tissues. For identifying expressed genes, our method achieved mean precisions from 0.89 to 0.94 and mean recalls between 0.89 and 0.90. Compared to the other methods, our algorithm provided a more stable and balanced performance, which is crucial for the discovery of candidate TAAs.

**Table 1:** Performance evaluation of the single-sample gene expression state inference algorithms

Tissue	Method	Non-expressed gene				Method	Expressed gene			
		Precision		Recall			Precision		Recall	
		Mean	SD	Mean	SD		Mean	SD	Mean	SD
Liver	TPM < 1	0.92	0.03	0.77	0.02	TPM > 1	0.95	0.01	0.87	0.02
	TPM < 2	0.83	0.05	0.80	0.01	TPM > 2	0.97	0.01	0.81	0.04
	zFPKM	0.99	0.00	0.45	0.04	zFPKM	0.78	0.01	0.96	0.00
	EnB	0.78	0.01	0.40	0.04	EnB	0.51	0.01	0.96	0.00
	variable	0.98	0.00	0.66	0.03	variable	0.90	0.02	0.90	0.00
Lung	TPM < 1	0.97	0.01	0.64	0.02	TPM > 1	0.89	0.01	0.89	0.00
	TPM < 2	0.94	0.02	0.70	0.02	TPM > 2	0.92	0.01	0.87	0.01
	zFPKM	0.99	0.00	0.45	0.04	zFPKM	0.78	0.01	0.96	0.00
	EnB	0.80	0.01	0.33	0.03	EnB	0.48	0.01	0.96	0.00
	variable	0.97	0.01	0.63	0.03	variable	0.89	0.01	0.89	0.00
Ovary	TPM < 1	0.97	0.01	0.70	0.03	TPM > 1	0.94	0.01	0.90	0.00
	TPM < 2	0.94	0.01	0.75	0.02	TPM > 2	0.96	0.01	0.88	0.01
	zFPKM	0.99	0.00	0.55	0.05	zFPKM	0.84	0.02	0.96	0.00
	EnB	0.78	0.04	0.41	0.10	EnB	0.53	0.03	0.96	0.00
	variable	0.96	0.01	0.71	0.03	variable	0.94	0.01	0.89	0.00
Pancreas	TPM < 1	0.84	0.05	0.74	0.02	TPM > 1	0.97	0.01	0.84	0.02
	TPM < 2	0.72	0.07	0.77	0.01	TPM > 2	0.98	0.01	0.78	0.05
	zFPKM	0.98	0.00	0.51	0.04	zFPKM	0.85	0.01	0.96	0.00
	EnB	0.68	0.03	0.46	0.06	EnB	0.53	0.02	0.96	0.00
	variable	0.94	0.01	0.66	0.02	variable	0.94	0.01	0.89	0.00

The algorithms included in this evaluation are the fixed threshold methods (TPM < 1, TPM < 2), zFPKM, EnB, and our single-sample gene expression state inference algorithm (variable). Mean refers to the average of the precision or recall rate for each sample, and SD is the standard deviation of precision or recall rate for each sample. TPM, transcripts per million. See also Table S1.

### Performance evaluation of tissue-level inference of non-expressed genes

**Figure 2:**
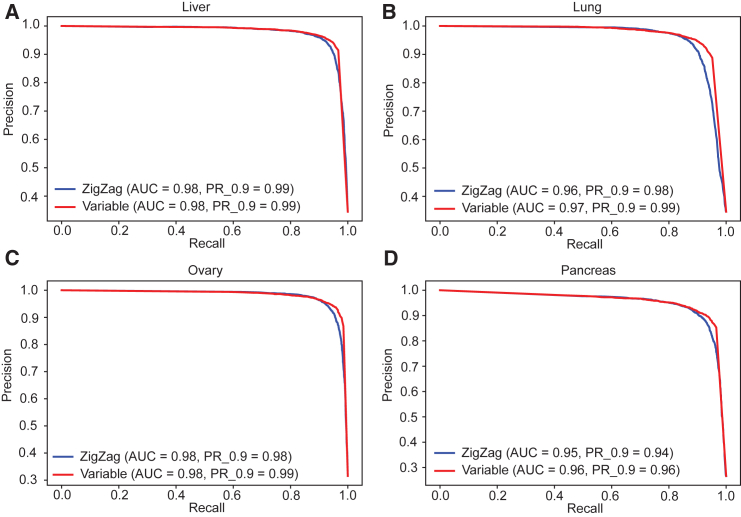
Comparison between the ZigZag method and our single-sample gene expression state inference algorithm Comparison of precision-recall (PR) curves between the ZigZag method (blue) and the single-sample gene expression state inference algorithm (red) at the tissue level for four normal tissues: (A) liver, (B) lung, (C) ovary, and (D) pancreas. The areas under the PR curve (AUPRCs) for the ZigZag method across the tissues are 0.98, 0.96, 0.98, and 0.95, respectively, while the AUPRCs for the single-sample gene expression state inference algorithm across the tissues are 0.98, 0.97, 0.98, and 0.96, respectively. Setting the non-expressed ratio at 0.9, the ZigZag method achieved precision rates of 0.99, 0.98, 0.98, and 0.94 across the tissues, whereas the single-sample gene expression state inference algorithm achieved precision rates of 0.99, 0.99, 0.99, and 0.96, respectively. See also Table S1.

Because dormant gene identification is determined at the tissue level, we further conducted a comparative analysis between our method and ZigZag, which was designed for tissue-level inference. We continued to use the reference standard derived from epigenomic data. In this analysis, our method produced precision-recall (PR) curves highly similar to those from the ZigZag method (Figure 2). The area under the PR curve (AUPRC) scores for our method were 0.98 for liver, 0.97 for lung, 0.98 for ovary, and 0.96 for pancreas. These scores were the same as or higher than those from ZigZag. When the non-expressed ratio was set at 0.9, our method achieved very high precision scores, with 0.99 for liver, lung, and ovary and 0.96 for pancreas. These scores were also the same as or higher than those from ZigZag (Figure 2). As highlighted earlier, achieving higher precision is paramount in predicting non-expressed genes. A noteworthy advantage of our method is its applicability at the single-sample level, a capability not achievable by ZigZag. This feature is particularly crucial for characterizing gene expression states in individual tumor samples, allowing for the identification of specific tumors with active TAA expression.

### Dormant gene identification from 54 distinct GTEx normal tissues

**Figure 3:**
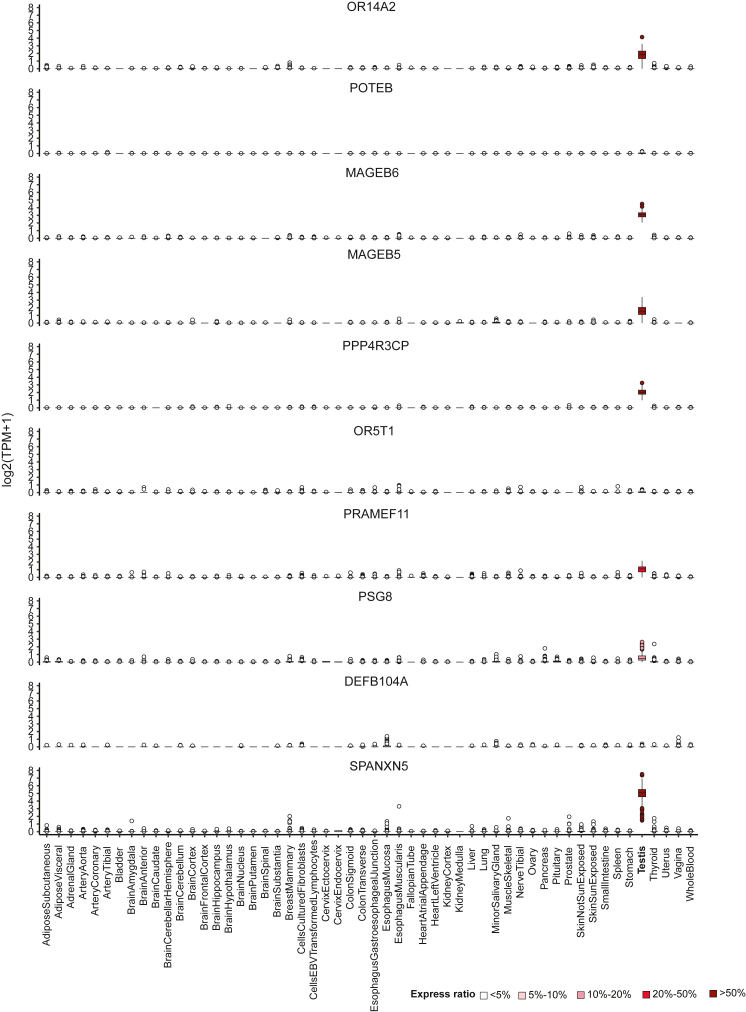
Expression patterns of 10 selected dormant genes across 54 normal tissues From top to bottom: OR14A2, POTEB, MAGEB6, MAGEB5, PPP4R3CP, OR5T1, PRAMEF11, PSG8, DEFB104A, and SPANXN5, with testicular sample expressed ratios of 72.24%, 0%, 100%, 60.06%, 97.45%, 0%, 32.29%, 11.90%, 0%, and 100%, respectively. See also Table S2.

To identify dormant genes in normal tissues, we obtained gene expression data from GTEx, comprising a total of 17,382 samples from 54 distinct normal tissues in TPM format, ranging from 4 to 803 samples per tissue. Applying our single-sample gene expression state inference method to these data and with a minimum tissue level non-expressed ratio of 0.9 across all tissue types except for testis, we identified a total of 909 dormant genes (Table S2). Figure 3 illustrates the expression patterns of 10 selected dormant genes, OR14A2, POTEB, MAGEB6, MAGEB5, PPP4R3CP, OR5T1, PRAMEF11, PSG8, DEFB104A, and SPANXN5, across all 54 kinds of normal tissues. Most of these genes were exclusively expressed in testis, with the exception of POTEB and OR5T1, which were not expressed in any normal tissues. Out of all 909 dormant genes identified, 291 (32%) exhibited testis-exclusive expression (with a testicular sample expressed ratio >10%), while the remaining 618 (68%) were not expressed in any normal tissues (Table S2). Therefore, all the identified dormant genes are promising candidates for further investigation to identify TAAs.

### TAA prediction across the 33 TCGA cancer types

**Figure 4:**
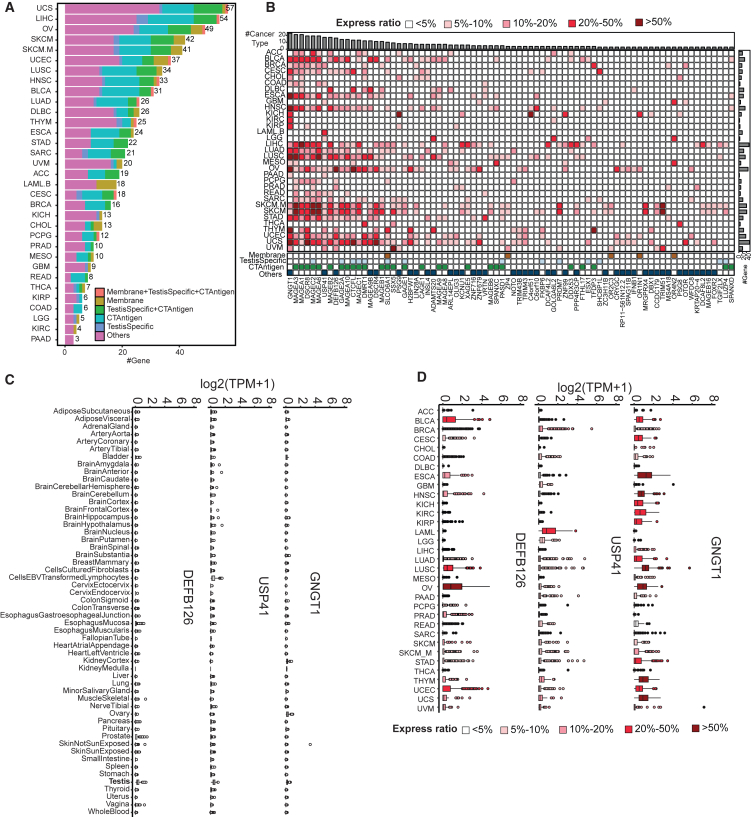
TAA prediction across the 33 TCGA cancer types (A) Bar plots depicting the number of 212 TAAs identified for each TCGA cancer type. Bar colors indicate cellular location and testis tissue expression level. (B) Heatmap displaying the expression ratio of 78 TAAs with a minimum 5% expressed rate of tumor samples in at least 3 cancer types out of the 33 TCGA cancer types. (C) Gene expression in log2(TPM+1) levels across 54 GTEx normal tissues of GNGT1, USP41, and DEFB126, respectively. (D) Gene expression in log2(TPM+1) levels across 33 TCGA cancer types of GNGT1, USP41, and DEFB126, respectively. See also Figure S1 and Table S3.

To predict candidate TAAs, we processed TCGA gene expression data for 33 cancer types utilizing the same protocol used for GTEx data analysis (STAR Methods). Next, we applied our single-sample gene expression state inference method to individual samples. Varying the expressed ratio cutoff in individual cancer types resulted in different numbers of TAA candidates across all cancer types (Figure S1). With the expressed ratio cutoff going from 1% to 50%, the number of TAA candidates decreased from 466 to 22. We selected a 5% cutoff to ensure that the identified TAAs could benefit a substantial portion of the cancer patient population. Among the 909 dormant genes identified from the GTEx data analysis, 212 showed an expressed state in at least 5% of tumors in one or more cancer types. These were designated as candidate TAAs. Across the 33 cancer types, uterine carcinosarcoma (UCS), liver hepatocellular carcinoma (LIHC), ovarian serous cystadenocarcinoma (OV), skin cutaneous melanoma (SKCM), metastatic skin cutaneous melanoma (SKCM.M), and uterine corpus endometrial carcinoma (UCEC) had the largest number of predicted candidate TAAs (Figure 4A). We further annotated the candidate TAAs based on the *in silico* human surfaceome,^23^ known CTAs,^11^ and the testis-specific protein list^24^ (STAR Methods; Table S3; Figure 4A). Notably, 39 genes are known CTAs, and another 12 encode testis-specific proteins. Moreover, 29 genes encode membrane proteins, which may serve as candidate targets for chimeric antigen receptor (CAR)-T and antibody-drug conjugates.

Among the 212 candidate TAAs, 78 were identified in three or more cancer types (Figure 4B). Interestingly, known CTAs, such as MAGEA3, MAGEA1, DSCR8, MAGEC2, and MAGEA6, tended to be identified in the largest number of cancer types. Nevertheless, we also recurrently identified some other candidate TAAs across many cancer types, such as GNGT1 in 23 cancer types, USP41 in 17 cancer types, and DEFB126 in 16 cancer types. As depicted in Figures 4C and 4D, these three genes exhibit no expression across all 54 normal tissues and display notable expression levels in multiple TCGA cancer types. This suggests the potential pan-cancer utility of these genes as TAAs. The list of 78 also included five encoding membrane proteins, namely SLCO6A1, ZP4, OR2C3, OR1N1, and OR4N2, which are potential pan-cancer targets for CAR-T and antibody-drug conjugates.

### Validation and prioritization of candidate TAAs

To assess the robustness of our prediction and prioritize the candidate TAAs for further experimental investigation, we conducted validation analysis using seven independent cancer cohorts with both tumor and normal RNA-seq and mass spectrometry proteomics data. These included hepatocellular carcinoma (HCC)^25^ and six cancer types from the CPTAC pan-cancer resource (https://proteomic.datacommons.cancer.gov/pdc/cptac-pancancer): UCEC, lung squamous cell carcinoma (LSCC), head and neck squamous cell carcinoma (HNSCC), lung adenocarcinoma (LUAD), clear cell renal cell carcinoma (CCRCC), and pancreatic ductal adenocarcinoma (PDAC).^26^^,^^27^

Based on RNA-seq data, the validation rates for each cohort were as follows: 48 out of 54 (89%) for HCC, 21 out of 37 (57%) for UCEC, 27 out of 34 (79%) for LSCC, 25 out of 33 (76%) for HNSCC, 17 out of 26 (65%) for LUAD, 3 out of 4 (75%) for CCRCC, and 1 out of 3 (33%) for PDAC (Table S4; Figure S2). Across these seven cancer types, we validated a total of 78 out of the 212 candidate TAAs identified from the 33 TCGA cancer types. All 78 candidate TAAs exhibited no expression across all 54 normal tissues except for testis (Figure S3). Moreover, proteomics detected 18 of these TAAs in the exact tumor tissues where they were inferred to be expressed based on RNA-seq data (Table S4; Figures S2 and S3).

**Figure 5:**
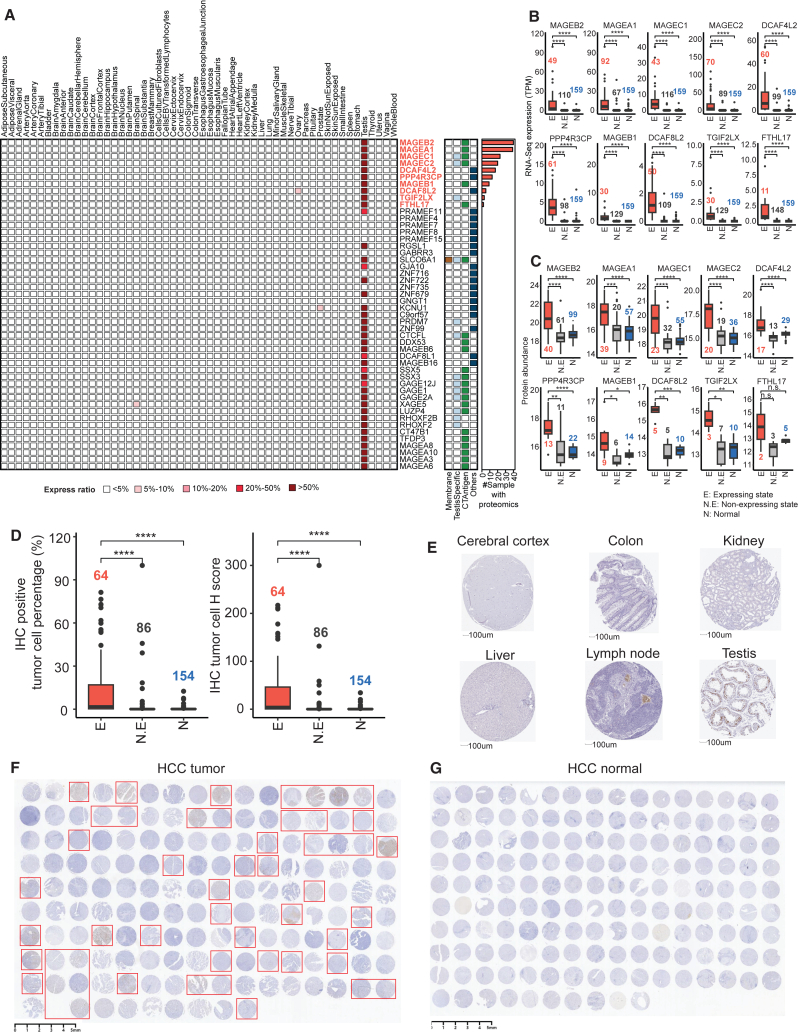
Validation and prioritization of liver cancer-associated antigen candidates (A) Heatmap depicting the tissue-level gene expression ratio across 54 GTEx normal tissues for the 48 candidate TAAs identified by both LIHC and HCC cohorts. The candidates include cellular location and testis tissue expression level annotations, with genes having paired samples proteomic evidence highlighted in red. (B) Boxplots comparing RNA-seq expression levels in TPM among inferred expressed samples, non-expressed samples, and normal samples. The numbers in color indicate the sample count for each group. The asterisks denote significance levels determined by the Wilcoxon signed-rank test. ∗*p* < 0.05, ∗∗*p* < 0.01, and ∗∗∗*p* < 0.001. (C) Proteomic expression levels are compared in a similar boxplot format, with significance levels also indicated by asterisks. (D) Additional boxplots detail comparisons of immunohistochemistry (IHC) positive tumor cell percentage and IHC tumor cell H-score for MAGEC2 among the three sample groups, with colored sample counts and significance levels as described. (E) IHC experimental results from the Human Protein Atlas (HPA) for MAGEC2 across 6 normal tissues, including cerebral cortex, colon, kidney, liver, lymph node, and testis. (F and G) IHC experimental results for MAGEC2 across HCC tumor (F) and adjacent normal liver (G), with the red square representing the positive staining results. See also Figures S2 and S3 and Tables S4 and S5.

LIHC is one of TCGA cancer types with the highest number of predicted TAAs (54 in total) and the largest number of validated TAAs in the independent cohort (48 in total) (Figures 4A and 5A). Importantly, 10 of these TAAs were detected by proteomics in the exact tumor tissues where they were inferred to be expressed based on RNA-seq data, including MAGEB2, MAGEA1, MAGEC1, MAGEC2, DCAF4L2, PPP4R3CP, MAGEB1, DCAF8L2, TGIF2LX, and FTHL17. By categorizing all 159 tumor samples in the validation cohort into expressed and non-expressed states for a particular gene, the expressed samples exhibited significantly higher expression levels compared to non-expressed samples and normal samples at both the mRNA (Figure 5B) and proteomics (Figure 5C) levels.

As an additional validation, we conducted immunohistochemistry (IHC) experiments on MAGEC2 using tissue blocks from the validation cohort (Figures 5D, 5F, and 5G). By comparing both IHC positive tumor percentage and IHC tumor H-score across the 150 HCC tumor and 154 normal samples, the expressed samples exhibited significantly higher positive levels compared to non-expressed samples and normal samples (Figure 5D; Table S5). The IHC experiments revealed a 32% positive staining rate across all HCC tumors (Figure 5F) and no positive staining signals across the paired normal samples (Figure 5G). Among the tumor samples with positive staining results, 82% (41/50) were inferred to express the MAGEC2 gene based on RNA-seq data. According to IHC experiments in the Human Protein Atlas (HPA) for normal tissues, positive signals were only observed in the testis but not in other normal tissues (Figure 5E). In summary, the 10 selected liver cancer TAAs represent promising candidates for further exploration as cancer therapeutic targets.

### Peptide warehouse for immunotherapy in liver cancer

**Figure 6:**
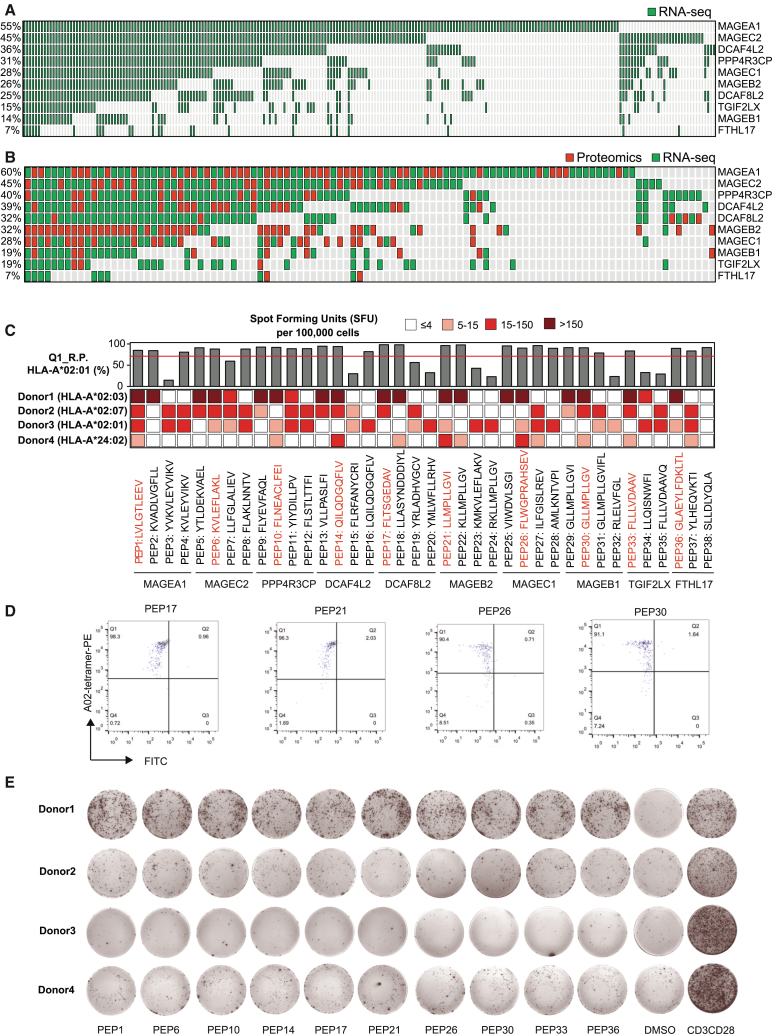
HCC-associated TAAs and TCR-T antigen identification with experimental validation (A and B) Distribution of LIHC-associated and HCC-associated TAAs across 369 LIHC cohorts and 159 HCC cohorts, respectively. Green dots represent inferred expressed in RNA-seq level, and red dots indicate the TAAs with proteomics evidence from matched samples. (C) Experimental validation of binding affinity and immunogenicity for 38 peptides with the highest binding affinities to the most common HLA-A02 allotype for the 10 prioritized proteins in (A) and (B). The bar plot illustrates the exchange efficiency of HLA-A02:01 tetramer quantified by Q1 replacement percentage. The red line indicates 70% replacement, the threshold for identifying a peptide with strong binding affinity. The heatmap depicts spot-forming units (SFUs) per 100,000 cells from ELISpot experiments. Red text highlights 10 peptides showing both strong exchange efficiency (>70%) and strong immunogenicity (SFUs >50), which are promising candidates for further investigation as broadly applicable immunotherapy targets. (D) Flow cytometry analysis results of the exchange efficiency for four selected peptides (PEP17, PEP21, PEP26, and PEP30). The scatterplots show the fluorescence intensities of A02-tetramer-PE (y axis) and fluorescein isothiocyanate (FITC; x axis) for each peptide. Each plot is divided into four quadrants: Q1 (high PE, low FITC), Q2 (high PE, high FITC), Q3 (low PE, high FITC), and Q4 (low PE, low FITC). The percentages indicate the proportion of beads in each quadrant. For all four peptides, more than 90% of the beads fall into Q1, indicating that the target peptides have efficiently exchanged into the MHC. (E) ELISpot images for 10 selected peptides in four individuals. See also Figure S4 and Table S6.

The 10 prioritized liver cancer TAAs showed RNA expression in 64% of samples in both TCGA cohort (Figure 6A) and the validation cohort (Figure 6B). In the validation cohort, among the tumor samples that showed TAA RNA expression, 72% also had matched evidence at the protein level, as indicated by proteomics analysis. To establish a peptide warehouse for the development of immunotherapy in liver cancer, we selected 38 peptides (Table S6) with computationally predicted binding affinities below 150 nM to the most common HLA-A02 allotype from the 10 prioritized liver cancer TAAs. We experimentally assessed the binding affinity of the 38 peptides through a competitive peptide binding assay (STAR Methods). The exchange efficiency of the HLA-A02:01 tetramer with all 38 selected peptides ranged from 15% to 98%, with 30 peptides exhibiting an exchange efficiency exceeding 50%, a predefined threshold indicating strong binding affinity (Figures 6C and S4). Subsequently, we investigated the immunogenicity of these peptides using an interferon (IFN)-gamma ELISpot assay (STAR Methods). In our experiments involving four donors, each one showed reactivity to some of these peptides (Figure 6C). Representative examples of peptides demonstrating robust exchange efficiency against HLA-A02 and exhibiting strong immunogenicity are illustrated in Figures 6D and 6E, respectively. Interestingly, CD8^+^ T cell cultures from donor 1 with HLA-A02:03 displayed potent reactivity against almost all selected peptides. In total, 21 peptides from all 10 prioritized liver cancer TAAs demonstrated both strong exchange efficiency (>70%) and robust immunogenicity (spot-forming units [SFUs] >50). These peptides represent promising candidates for further investigation as potential immunotherapy targets.

## Discussion

In this study, we developed a single-sample gene expression state inference algorithm, which was applied to RNA-seq data from the GTEx and TCGA databases, uncovering 212 candidate TAAs spanning 33 cancer types. Notably, 78 of these TAAs were found to be expressed in 5% or more tumors across at least three cancer types. Validation across seven independent cancer types confirmed 78 out of the 212 TAAs predicted from 33 TCGA cancer types. Specifically, 48 of the 54 TAAs predicted from TCGA liver cancer data were validated in an independent liver cancer cohort, with 10 further corroborated through proteomics. These 10 TAAs showed expression in about 64% of the samples in both TCGA and independent cohorts. From these, 38 peptide sequences were identified with a high predicted binding affinity to HLA-A02, and subsequent experimental validation revealed significant binding affinity and immunogenicity for HLA-A02 in 21 peptides, highlighting their potential as promising candidates for peptide vaccine or TCR-T cell-based cancer immunotherapies in liver cancer. These results underscore the power of integrating computational and experimental methodologies to identify and validate novel targets for cancer immunotherapy.

A key strength of our method lies in the robust inference of gene expression states at the single-sample level, evaluated on RNA-seq data from four normal tissues with epigenomic evidence. In terms of single-sample gene expression state inference, our variable threshold method demonstrated greater stability and balanced performance compared to existing approaches. At the tissue level, our method performed similarly to the ZigZag method, which was specifically developed for tissue-level inference.

### Limitations of the study

One limitation of our study is the stringent requirement of non-expression in all normal tissues except for testis. It is acknowledged that, in practical therapeutic applications, allowing a certain level of expression in normal tissues may be acceptable. Future research efforts can focus on algorithm optimization to provide different lists of candidate TAAs at varying expression levels, facilitating clinical research applications. Furthermore, the 21 peptides we identified for liver cancer warrant further pre-clinical and clinical experimental validation. Additionally, since our experimental validation was only performed for liver cancer, future research could be extended to the other six cancer types.

## Resource availability

### Lead contact

Further information and requests for resources and reagents should be directed to and will be fulfilled by the lead contact, Bing Zhang (bing.zhang@bcm.edu).

### Materials availability

This study did not generate new unique reagents.

### Data and code availability

• This paper analyzes existing, publicly available data. The accession numbers for these datasets are listed in the key resources table. Other result files are available at Zenodo (https://zenodo.org/records/13948975).
• The source code of expression state Bayesian inference for each sample and tissue expression state inference is freely available at Zenodo (https://doi.org/10.5281/zenodo.13948975).
• Any additional information to reanalyze the data reported in this paper is available from the lead contact upon request.

## Acknowledgments

This study was supported by the 10.13039/100000002National Institutes of Health (NIH) through grants from the 10.13039/100000054National Cancer Institute (NCI) (R01 CA245903 and U24 CA271076, both to B.Z.), funding from the McNair Medical Institute at The Robert and Janice McNair Foundation (to B.Z.), and grants from the 10.13039/501100001809National Natural Science Foundation of China (22304111 to X.Y., 82130077 and 82121002 to Q.G., and 32170664 to J.L.). Additional support was provided by the Shanghai Pujiang Program (23PJ1406400 to X.Y.), the Startup Fund for Young Faculty at SJTU (AF0800094 to X.Y.), and the Key Project for Computational Biology of Shanghai (23JS1400800 to J.L.). B.Z. is a McNair Scholar.

## Author contributions

Conceptualization, B.Z.; methodology, X.Y. and B.Z.; formal analysis, X.Y. and B.Z.; investigation, X.Y., B.Z., L.D., J.L., and Y.D.; validation, H.Z. and S.H.; writing – original draft, B.Z. and X.Y.; supervision, Q.G. and B.Z.; funding acquisition, X.Y., J.L., Q.G., and B.Z.

## Declaration of interests

B.Z. received research funding from AstraZeneca and consulting fees from Inotiv.

## STAR★Methods

### Key resources table

**Table:** 

REAGENT or RESOURCE	SOURCE	IDENTIFIER
**Antibodies**		

MAGEC2	Abcam	Abcam Cat#ab209667
HRP conjugated Goat Anti-Rabbit IgG	Abcam	Abcam Cat#ab97051; RRID: AB_10679369

**Biological samples**		

PBMCs isolated from peripheral blood of healthy donors	This paper	Zhongshan Hospital, Fudan University
Tissue microarray of HCC patients	Gao et al.^25^	Zhongshan Hospital, Fudan University

**Chemicals, peptides, and recombinant proteins**		

Peptides	China Peptides Inc.; Genemed Synthesis Inc.	N/A
Lymphoprep	StemCell	Catalog: 07851
ImmunoCult-XF T cell expansion medium	StemCell	Catalog: 10981
ImmunoCult Human CD3/CD28 T cell Activator	Stemcell	Catalog: 10971
Human recombinant IL-2	Stemcell	Catalog: 78036

**Critical commercial assays**		

QuickSwitch Quant HLA-A∗02:01 Tetramer Kit-PE	MBL International	Catalog: TB-7300-K1
IFN-g ELISpot kit	DAKEWE	Catalog: 2110003

**Deposited data**		

GTEx normal tissue RNA-seq data	GTEx Portal	https://gtexportal.org/home/
TCGA Pan-Cancer RNA-seq data	Genomic Data Commons Data Portal	https://portal.gdc.cancer.gov/
CPTAC Pan-Cancer RNA-seq data and mass spectrometry data	CPTAC pan-cancer resource	https://proteomic.datacommons.cancer.gov/pdc/cptac-pancancer
HCC RNA-seq data and mass spectrometry data	Gao et al.^25^	https://pubmed.ncbi.nlm.nih.gov/31585088/
Epigenomic data	The Roadmap Epigenomics Project	https://egg2.wustl.edu/roadmap/web_portal
512 testicular proteins list	Pineau et al.^24^	https://pubs.acs.org/doi/10.1021/acs.jproteome.9b00351
CT Database	Almeida et al.^11^	http://www.cta.lncc.br/
Membrane proteins	Damaris et al.^23^	https://pubmed.ncbi.nlm.nih.gov/30373828
IHC experimental results from the Human Protein Atlas	Human Protein Atlas Portal	https://www.proteinatlas.org/

**Software and algorithms**		

mixtools v2.0.0	Benaglia et al.^28^	https://cran.r-project.org/web/packages/mixtools/index.html
ZigZag v0.1.0	Thompson et al.^21^	https://github.com/ammonthompson/zigzag
NetMHCpan v4.0	Hoof et al.^29^	http://www.cbs.dtu.dk/services/NetMHCpan-4.0/
RNA-SeQC v2.3.5	DeLuca et al.^30^	https://github.com/getzlab/rnaseqc
ComplexHeatmap v2.15.4	Gu et al.^31^	https://bioconductor.org/packages/release/bioc/html/ComplexHeatmap.html
Qupath v0.5.1	Bankhead et al.^32^	https://qupath.github.io/
TAA prediction source code v1.0	This paper	https://doi.org/10.5281/zenodo.13948975

### Experimental model and study participant details

Peripheral blood mononuclear cells (PBMCs) from healthy donors were isolated at Zhongshan Hospital, Fudan University using Lymphoprep (07851, Stemcell) and cultured with ImmunoCult-XF T cell Expansion Medium (10981, Stemcell) supplemented with human recombinant IL-2 (78036, Stemcell). ImmunoCult Human CD3/CD28 T cell Activator (10971, Stemcell) was added to the cell suspension, which was then incubated for 3 days. Subsequent expansion of the cell suspension was carried out every two days as per manufacturer’s instructions. The entire process was approved by the Institutional Review Board of Zhongshan Hospital, Fudan University (B2024-412).

### Method details

#### Gene expression state Bayesian inference for each sample

The probability density function (pdf) of RNA-seq expressions for a collection of genes from one sample is denoted as $f\left(x\right)=\pi_{ne}f_{ne}\left(x\right)+\pi_{e}f_{e}\left(x\right)$. Here, $f_{ne}\left(x\right)$ represents the pdf of expressions for not expressed genes, $f_{e}\left(x\right)$ represents the pdf of expressions for expressed genes, $\pi_{ne}$ is the proportion of not expressed genes, and $\pi_{e}=1-\pi_{ne}$. The probability of a gene being expressed in the sample is given by Bayesian rule:

$$\text{Prob}\left(\text{Expressed}|X=x\right)=\left\{ \begin{array}{c} 0,if\;x=0\\ \frac{\pi_{e}f_{e}\left(x\right)}{\pi_{ne}f_{ne}\left(x\right)+\pi_{e}f_{e}\left(x\right)},\text{otherwise} \end{array}\right.$$

The expression state of a gene in the sample can be defined using a probability cutoff of 0.5:

$$\text{Expression}\;\text{state}=\left\{ \begin{array}{c} 1,if\;\text{Prob}\left(\text{Express}|X=x\right)\geq 0.5\\ 0,if\;\text{Prob}\left(\text{Express}|X=x\right)<0.5 \end{array}\right.$$

#### Parameter estimation

To estimate $f_{ne}\left(x\right)$, $f_{e}\left(x\right)$, $\pi_{ne}$ and $\pi_{e}$, we assume that the RNA-seq expression distribution of all genes in one sample can be separated into two distinct lognormal mixture distributions based on their expression abundance.^19^^,^^20^ The Expectation Maximization (EM) algorithm, implemented in the R package "mixtools",^28^ is employed for parameter estimation. Due to the EM algorithm’s dependence on initial values, we leverage housekeeping genes to aid in selecting appropriate initial values.

#### Tissue expression state inference

At the tissue level, the expression state of a gene *i* in tissue *j* is defined as follows: gene *i* is considered not expressed in tissue *j* if it does not express in a predefined proportion of samples from tissue *j.* Let tissue *j* contains *m* samples, and ${\text{Expression}\;\text{state}}_{n}$ indicate the expression state of gene *i* in sample *m* from tissue *j.* The not expressed ratio of gene *i* in tissue *j* is defined as:

$$\text{Ratio}\left(\text{gene}\;i\;\text{not}\;\text{expressed}\;\text{in}\;\text{tissue}\;j\right)=\frac{\sum_{n=1}^{m}\left(1-{Expression\;state}_{n}\right)}{m}$$

In this manuscript, we used 0.9 as the ratio cutoff to define gene *i* as not expressed in tissue *j*.

#### RNA-sequencing data quantification for TCGA and HCC

We leveraged RNA-seq data from TCGA and HCC datasets to identify TAAs. For TCGA, our comprehensive analysis covered 33 distinct cancer types. Our gene-level expression quantification was conducted using RNA-SeQC v2.3.5^29^ with the annotation of Gencode 26 for both datasets. Recognizing the limitations of RNA-SeQC in handling full annotation gtfs, we implemented a Python script sourced from the RNA-SeQC GitHub repository (https://github.com/getzlab/rnaseqc/issues/34) to collapse the gene annotations. This approach ensured a robust and standardized analysis across all genes, contributing to the reliability of our results.

#### Epigenomic data utilized for defining gene expression state in normal tissues

The Roadmap Epigenomics Project^22^ employed a machine learning approach to categorize 15 gene states based on chromatin epigenetic marks. Previous studies indicate that active genes exhibit three key features.

(1) Possession of at least one active promoter or promoter-flanking mark overlapping with its exon sequences (states 1 and 2).
(2) Presence of at least one active transcription mark (states 3, 4, 5).
(3) Absence of marks associated with repressed expression (states 9–15).

Conversely, inactive genes exhibit two distinctive features:

(1) Lack of active promoter or transcription marks (states 1–5).
(2) Presence of at least one mark associated with repressed expression (states 9–15).

We applied this classification method to analyze four normal tissues: pancreas, liver, lung, and ovary.

#### Identification of testis-specific proteins

We downloaded a list of 512 testicular proteins identified using genome-wide transcriptomic analysis and Immunohistochemistry in a recent publication.^24^ Next, we downloaded RNA-Seq data from GTEx for all 54 normal tissues and manually checked the RNA-Seq expression levels of the 512 testicular proteins across all normal tissues. Proteins with obvious mRNA expression in non-testis tissues were removed, resulting in a list of 180 testis-specific proteins.

#### Membrane proteins

Cell surface proteins were acquired from the in silico surfaceome Table S3.^23^ Proteins with the label ‘pos. trainingset’ were selected. These proteins were present in at least two of three datasets: the Cell Surface Protein Atlas (high confidence), Uniprot “cell membrane” keyword, and high-confidence plasma membrane proteins in the COMPARTMENTS database.

#### IHC assay

Tissue microarrays (TMA) were obtained from an HBV related HCC cohort containing 154 HCC patients’ samples.^25^ Clinical sample acquisition was approved by the Research Ethic Committee of Zhongshan Hospital (B2024-412). Slides were deparaffinized in xylene and rehydrated. Antigen retrieval was performed in the microwave using Tris-EDTA buffer (PH = 9.0). Slides were cooled to ambient temperature and treated with 3% H2O2 solution for 10 min. After three times washes TMA were blocked with 3% BSA in PBS for 10 min and incubated at 4°C overnight with rabbit anti-human MAGEC2 antibody (ab209667, Abcam, 1:1000), HRP conjugated Goat Anti-Rabbit IgG antibody (ab97051, Abcam, 1:500) was subsequently stained at room temperature for 30 min. Slides were washed and counterstained with hematoxylin. Positive rate and IHC score of tumor and adjacent normal liver cells were calculated using Qupath software (version 0.5.1).^30^

#### Peptide-MHC tetramer exchange assays

We selected 38 peptides (Table S6) with NetMHCpan^31^ predicted binding affinities below 150nM to the most common HLA-A02 allotype from the 10 prioritized liver cancer TAAs. We synthesized these peptides following standard procedures. Peptides were loaded onto QuickSwitch Quant HLA-A02:01 (PE labeled) tetramers (TB-7300-K1, MBL International) and analyzed using a flow cytometer (BD LSRFortessa) to assess peptide exchange efficiency. Each peptide, including a reference peptide, was dissolved in DMSO and mixed with HLA-A02:01. After overnight incubation, Magnetic Capture Beads were added, and FITC-labeled Exiting Peptide Antibody was applied. By measuring the percentage of original peptide replaced by a competing peptide, we evaluated exchange efficiency to determine tetramer suitability for staining. Analysis settings were adjusted for optimal bead and control event detection.

#### ELISPOT assay

ELISPOT plates (2110003, DAKEWE) were pretreated with PBS before dispensing cell suspensions (nearly 1×10^5^ cells) with synthesized peptide (10 μg/mL) into each well. After incubation at 37°C in a CO_2_ incubator for 20 h, plates were washed and incubated with anti-human IFN-γ followed by Streptavidin-HRP. Staining of Streptavidin-HRP was performed using AEC solution, and the reaction was stopped by rinsing with cold tap water. ELISPOT plates were scanned and counted using an ImmunoSpot plate reader and associated software from Cellular Technologies, Ltd.

### Quantification and statistical analysis

For differential expression analysis among expressed, non-expressed, and normal samples for a given gene, *p*-values were calculated using the Wilcoxon rank-sum test. Unless otherwise specified, all statistical analyses were performed using R. Additional details are provided in the results and figure legends. Other statistical analyses related to gene expression state inference are described in the method details section.

## Supplemental information

Document S1. Figures S1–S4

Table S1. Classification of activated and inactivated genes by chromatin epigenetic marks in liver, lung, ovary, and pancreas from the Roadmap Epigenomics Project, related to Figure 2 and STAR Methods

Table S2. List of 909 dormant genes identified across 54 normal tissues, excluding testis, by single-sample gene expression state inference, related to Figure 3

Table S3. List of 212 TAAs across 33 TCGA cancer types using the TAA prediction and prioritization workflow, related to Figures 4 and S1

Table S4. Validation of candidate TAAs predicted from TCGA in seven independent cancer cohorts, related to Figures 5, S2, and S3

Table S5. Results of IHC experiments on MAGEC2 in tissue samples from the validation cohort, related to Figure 5 and STAR Methods

Table S6. Binding and IFN-gamma ELISPOT assays of 38 selected peptides from 10 prioritized liver cancer TAAs targeting the HLA-A02 allotype, related to Figures 6 and S4 and STAR Methods

Document S2. Article plus supplemental information
