# Supplementary material for: Tumor-associated antigen prediction using a single-sample gene expression state inference algorithm
Source: Cell Rep Methods. 2024 Nov 18;4(11):100906. doi: 10.1016/j.crmeth.2024.100906 (PMC11705763; doi:10.1016/j.crmeth.2024.100906)
Supplement: Document S1. Figures S1–S4 [file mmc1.pdf]

Cell Reports Methods, Volume *4*

Supplemental information

Tumor-associated antigen prediction using a single-sample gene expression state inference algorithm

Xinpei Yi, Hongwei Zhao, Shunjie Hu, Liangqing Dong, Yongchao Dou, Jing Li, Qiang Gao, and Bing Zhang

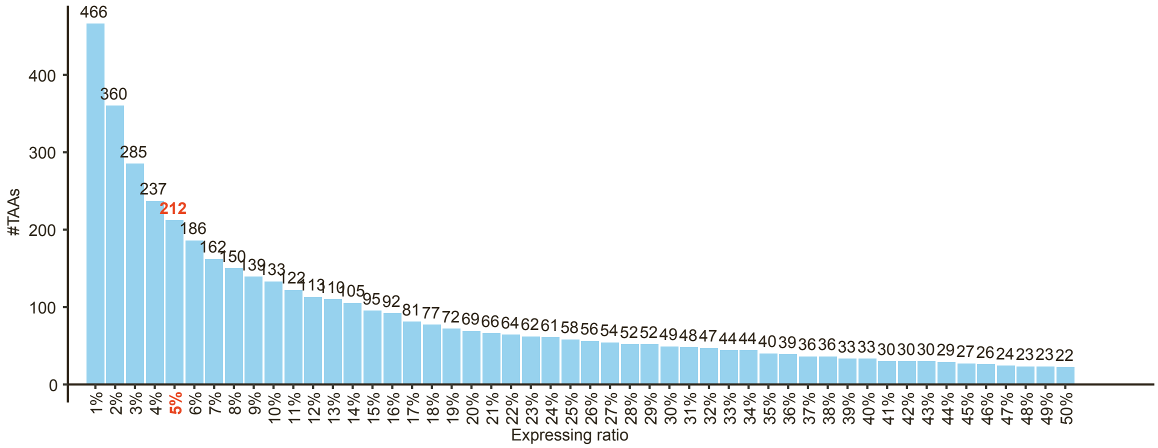

Figure S1. Impact of varying expressed ratios on the number of TAA candidates identified across 33 cancer types from TCGA. The x-axis represents the expressed ratio cutoff, ranging from 1% to 50%. The y-axis shows the corresponding number of TAA candidates identified, Related to Figure 4.

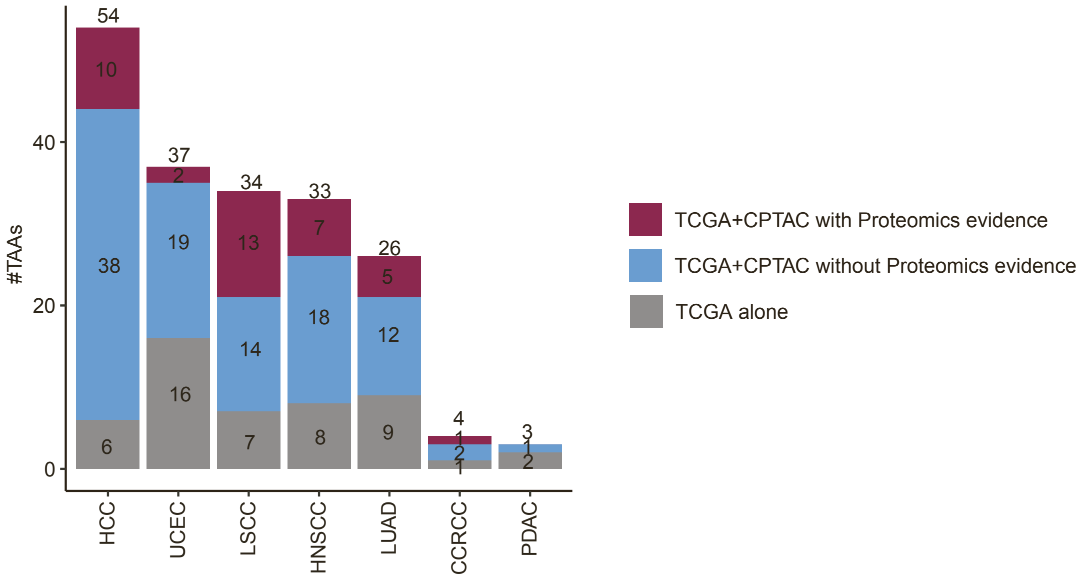

Figure S2. Validation of candidate TAAs identified from TCGA data across seven independent cohorts. The x-axis represents the different cancer types, and the y-axis shows the number of TAAs validated in the corresponding cohort. Each bar is divided into segments indicating the total number of TAAs identified, the number of TAAs validated by RNA-seq data, and the number of TAAs further validated by proteomics data, Related to Figure 5.

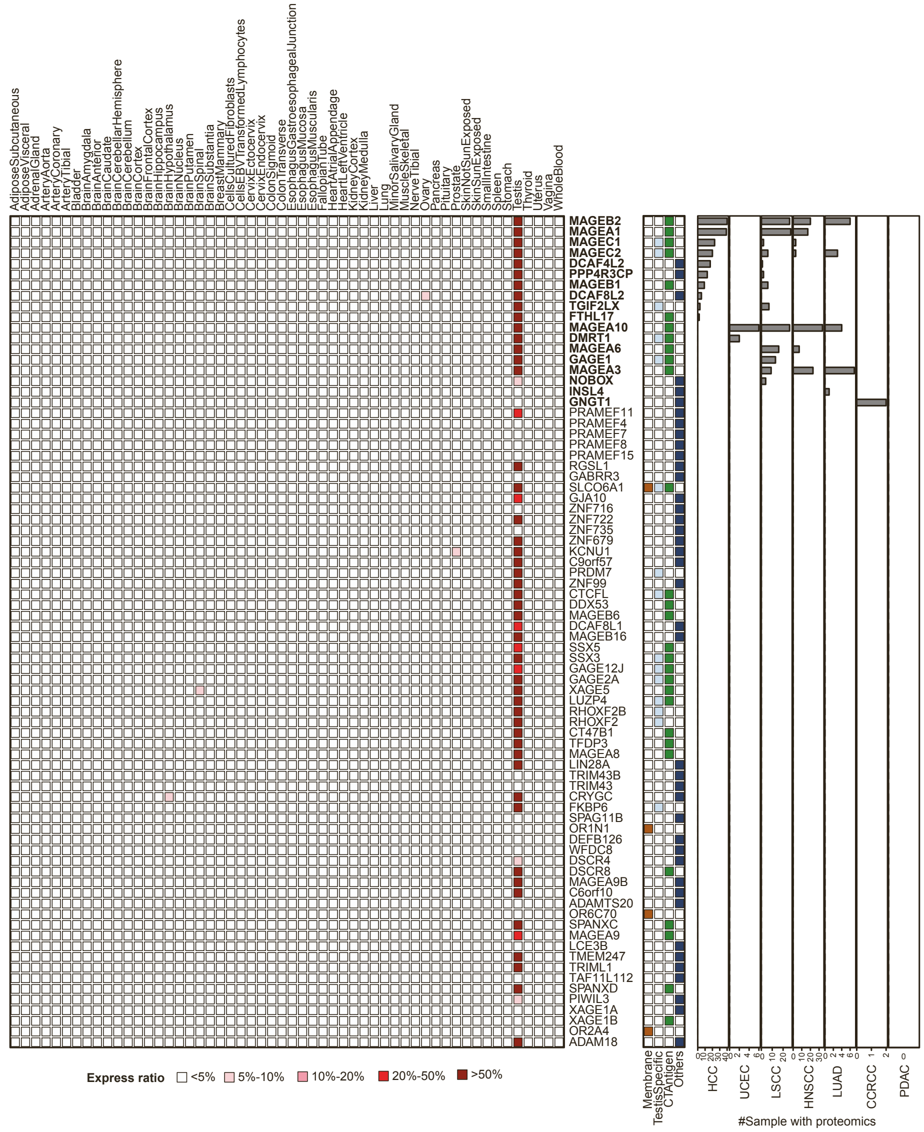

Figure S3. Expression patterns of 78 candidate TAAs in 54 GTEx normal tissues and 7 independent tumor cohorts. Heatmap depicting the tissue-level gene expression ratio across 54 GTEx normal tissues for the 78 candidate TAAs identified by both TCGA and 7 different independent tumor cohorts, including HCC, UCEC, LSCC, HNSCC, LUAD, CCRCC and PDAC. The candidates include cellular location and testis tissue expression level annotations, with genes having paired samples proteomic evidence highlighted in Bold, Related to Figure 5.

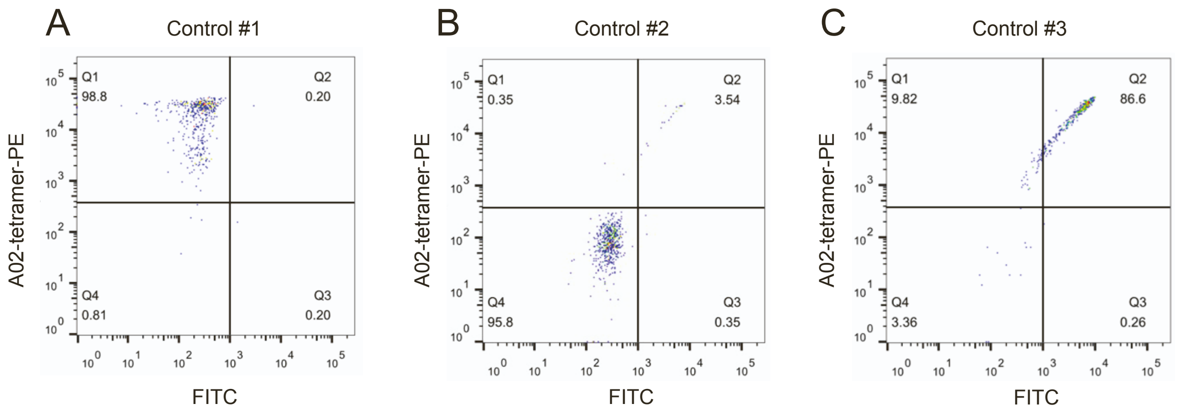

Figure S4. Control assays for the peptide exchange experiment. Three control samples were utilized to verify the assay's reliability. (A) Control #1: Represents the condition where an externally added peptide of interest successfully replaces the pre-bound peptides on HLA tetramers. The low FITC signal observed indicates successful peptide exchange or the absence of peptide, establishing the fluorescence level representative of complete peptide exchange. This serves as a positive control. (B) Control #2: Corresponds to the "Beads Only" condition, lacking both the pre-bound peptide and HLA tetramer. It is used to set the baseline fluorescence, showing minimal signal in both FITC and PE channels. (C) Control #3: Uses beads containing both the pre-bound peptide and HLA tetramer. The FITC signal indicates the presence of the pre-bound peptide or the failure of peptide exchange, providing a negative control. The quadrants Q1, Q2, Q3, and Q4 represent different populations based on their FITC and A02-tetramer-PE signals, with the percentage of each population indicated within the plots, Related to Figure 6 and STAR Methods.
